# Tuning of defect-mediated AgInS_2_-based chalcogenides for highly efficient electroactive energy storage electrodes

**DOI:** 10.1039/d6ra09800b

**Published:** 2026-10-22

**Authors:** Faiza Mustafa, Aneeqa Masood, Waris Ali, Ume Zainab, Ayesha Iram, Sameen Ilyas, Zhouyang Xiang, Ishrat Sultana, Aamir Razaq

**Affiliations:** a Department of Physics, COMSATS University Islamabad Lahore Campus 54000 Pakistan isultana@cuilahore.edu.pk aamirrazaq@cuilahore.edu.pk; b State Key Laboratory of Advanced Papermaking & Paper-based Materials, South China University of Technology Guangzhou 510640 China

## Abstract

Metal chalcogenides with enhanced electrochemical and electrical properties are promising next-generation supercapacitor electrodes. Cu-, Mn-, and Cu–Mn-doped and pristine AgInS_2_ nanoparticles have been synthesized through a simple and low-cost hydrothermal route. Herein, we demonstrate for the first time a direct correlation between dual-dopant synergy and the defect landscape and charge-transfer pathways of an AgInS_2_ lattice (I–III–VI_2_) that tolerates cation substitution without structural degradation. The structural analysis shows that both pristine and doped samples form a mixed orthorhombic/tetragonal AgInS_2_ framework with orthorhombic phase dominance, whereas doping reduces the crystallite size from ∼25 nm to ∼14, ∼9, and ∼18 nm for Cu-, Mn-, and Cu–Mn-doped samples, consistent with the SEM particle-size reduction from 40–55 nm in pristine AgInS_2_. Raman and X-ray photoelectron spectroscopy (XPS) verify the Ag–S and S–S vibrational modes and the Ag^+^, In^3+^ and S^2−^ chemical states of the host lattice, with Cu and Mn incorporated as Cu^+^ and Mn^2+^. UV-vis and photoluminescence (PL) spectroscopy indicate that doping modifies the electronic structure and defect landscape: the optical band gap varies from 3.52 eV to 3.50, 3.55 and 3.40 eV for the Cu-, Mn- and Cu–Mn-doped samples, while broad defect-related emission over 500–900 nm confirms intrinsic and dopant-induced defect states opening multiple charge-transfer pathways. The specific capacitance increases from 72.3 to 96.7, 129.8 and 189.27 F g^−1^ for the pristine and Cu-, Mn- and Cu–Mn-doped electrodes at 5 mV s^−1^, the capacitive contribution increases from 0.41% to 10.41%, 13.54% and 19.85%, and the charge-transfer resistance decreases from 1.652 Ω to 1.3, 1.22, and 0.693 Ω, with b-values of 0.5–0.69 indicating a hybrid diffusion and capacitive-controlled charge-storage mechanism. The improved performance of the co-doped electrode is ascribed to the synergistic effect of Cu and Mn, which enhances the accessibility of electroactive sites, interfacial charge transfer, and ion transportation, indicating that Cu–Mn co-doping is a promising strategy for defect and composition engineering of high-performance AgInS_2_-based supercapacitor electrodes.

## Abstract 2

Cu–Mn, co-doping modifies the defect structure of AgInS_2_, where intrinsic and dopant-related states could potentially facilitate charge transfer for energy storage applications.

## Graphical abstract

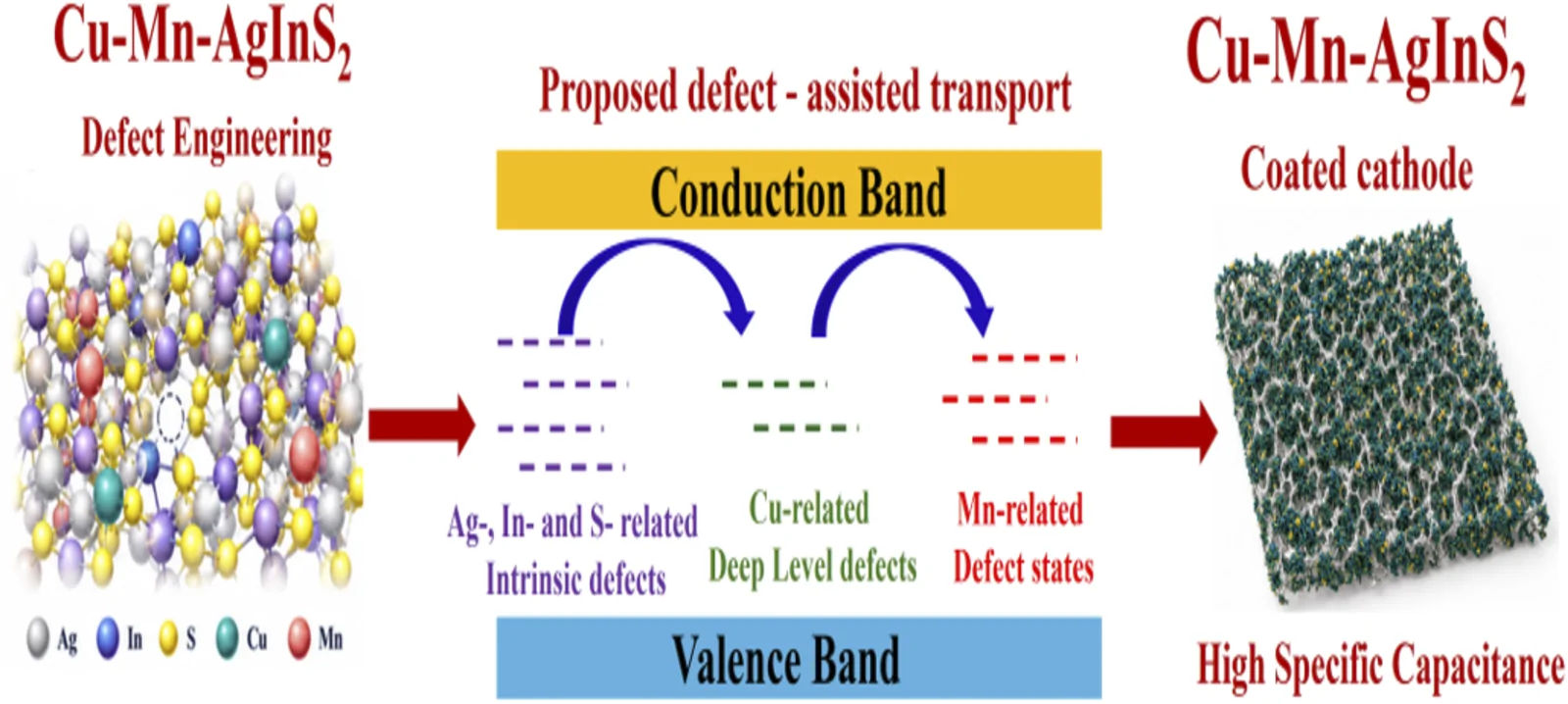

## 1. Introduction

The growing need for high-performance energy-storage technologies that is motivated by portable electronics, electric transportation, grid stabilization, and wearable systems, has heightened interest in finding new advanced electrode materials that can provide high energy as well as power density.^1,2^ Electrochemical storage systems, like batteries and supercapacitors (SCs), are considered promising options for supporting renewable energy systems.^3^ Supercapacitors have gained substantial scientific interest as energy storage devices owing to their ability to generate high power pulses, quick charge–discharge rates, and prolonged cycle life, but their comparatively low energy density relative to batteries is also a serious limiting factor.^4,5^ To overcome this shortcoming, electrode materials with both fast and reversible redox dynamics and high electrical conductivity, along with structural stability, are needed. Rational materials design has therefore been at the center of next-generation supercapacitor technologies development.^6,7^

Chalcogenides have emerged as potential electrode materials for the efficient functioning of next-generation energy storage devices, including batteries and supercapacitors.^8^ Transition metal chalcogenide's high conductivity, minimal internal resistance, enhanced stability, and quantum-sized effects are responsible for their better electrochemical performance when compared to the widely utilized metal oxides and carbon equivalents.^9,10^ The relatively covalent character of the metal–sulfur bonding is helpful to increase the charge-transfer kinetics and enhance the electrochemical reversibility.^11,12^ Specifically, ternary metal chalcogenides (TMCs) have clear benefits over binary materials by allowing compositional control over electronic structure, carrier concentration, and defect chemistry. The availability of several cations of different oxidation states dramatically enhances the density of electroactive sites as well as the range of faradaic reactions, making it conducive to high-rate pseudocapacitive behavior.^13^ Although these have such benefits, the real performance is usually hampered by interfacial resistance of charge-transfer as well as slow kinetics of ion-diffusion, particularly on high-rate operations.^14^ Dopant engineering is hence an effective approach to maximize ternary chalcogenides. Addition of aliovalent or redox-active metal ions has the potential to control band structure, add defect states, increase electrical conductivity, and speed up charge-transfer reactions.^15^ Copper incorporation is also known to increase the speed of electron moving, and decrease charge-transfer resistance, and manganese incorporation provides a variety of accessible oxidation states, which improve pseudocapacitive redox activity.^16,17^ More to the point, recent reports indicate that co-doping solutions have the potential to create synergies as they can enhance both electronic movement channels and ion-diffusion rates at the same time.^18^ Nonetheless, systematic studies involving dual-dopant effects in structurally stable ternary sulfide systems have not been performed, especially in Ag–In–S systems.

TMCs exhibit unique compositional flexibility and synergistic interactions among metal ions and provide abundant active sites for rapid charge transfer, leading to enhanced specific capacitance, superior rate capability, and long cycling life. Silver indium sulfide (AgInS_2_), an I–III–VI_2_ TMCs having a tunable lattice and compositional versatility, is a promising electrode material. Its multi-cation architecture allows controlled substitution with minimal lattice collapse, which allows electrochemical properties to be targeted.^19^

Carbon nanomaterials and hybrid energy-storage materials have been widely studied to address inherent limitations of supercapacitors, especially their comparatively low energy density. In this regard, carbon nanomaterials with modified surface chemistry and interconnected pathways for ion and electron transport have emerged as promising candidates for the enhancement of supercapacitors energy storage performance and electrochemical stability.^20^

Similarly, spherical carbon structures by using hydrogen-bond-assisted assembly are developed with interconnected active sites possessing a high energy density of approximately 166 Wh kg^−1^ with long-term cycling stability of 500 000 cycles.^21^ Study reveals incorporation of 3-dimensional carbon nanostructures having porous architecture in zinc-ion hybrid capacitors that provided structural stability and interconnected ion-transport pathways, resultantly achieving high energy density of 152 Wh kg^−1^ and cycling stability about 300 000 cycles.^22^

These studies revealed that tailored morphology, optimized surface functionality, power density and electrode–electrolyte interaction can be optimized in other metal composites like transition metal chalcogenides. Transition-metal chalcogenides offers additional redox-active sites and tunable electronic structure as compared to carbon nanomaterials that plays a significant role in pseudocapacitive charge storage mechanism. Among other transition-metal chalcogenides (TMCs), doped and multicomponent based chalcogenides offers additional opportunities like tunable electronic structure, more active sites and better ion-storage kinetics. Although considerable progress has been made in the development of transition-metal chalcogenides-based electrodes for electrochemical energy storage systems, the development in multicomponent TMCs-based electrodes for supercapacitor applications has not been sufficiently explored. Copper doped or manganese doped AgInS_2_ and their optical, electrical, and structural properties have been reported but to the best of our knowledge, synergistic integration of Cu and Mn into AgInS_2_ for its application in energy storage devices has not been studied. This work correlates the structural and electronic modification induced by the dopant with the measured electrochemical response and aims to demonstrate that Cu–Mn co-doping is an effective defect- and composition-engineering route for high-performance AgInS_2_ -based supercapacitor electrodes, and to provide a rational design basis for multication metal chalcogenide electrodes. Therefore, Cu–Mn–AgInS_2_ based electrode materials provide a novel approach for the improvement in electrochemical energy-storage performance by optimizing their composition and structural characteristics.

## 2. Experimentation

### 2.1 Reagents

All the chemicals were used directly as purchased without further purification. Silver nitrate (Ag(NO_3_), 99.8%, DUKSAN), indium chloride (InCl_3_, 98%, Sigma-Aldrich), thiourea (CH_4_N_2_S, Sigma-Aldrich), thioglycolic acid (TGA, Sigma-Aldrich), manganese nitrate trihydrate (Mn(NO_3_)_2_·3H_2_O >97%), Sigma-Aldrich), copper acetate (Cu(CH_3_COO)_2_·H_2_O, UNI-CHEM), potassium hydroxide (KOH, Sigma-Aldrich).

### 2.2 Synthesis of nanoparticles

The AgInS_2_ nanoparticles were synthesized *via* a facile hydrothermal route. The 0.2 mmol solution of Ag (NO_3_)_2_ and 0.1 mmol of InCl_3_ were dissolved in 50 mL of DI water in the presence of 2 mL of thioglycolic acid (TGA) stirred until complete dissolution. 0.2 mmol of thiourea was dissolved separately into a beaker containing 50 mL of DI water. Then, both solutions were mixed to form a homogeneous solution, transferred into a Teflon autoclave, and heated in an oven at 140 °C for 5 hours to obtain the fine precipitates of AgInS_2_. The precipitates were centrifuged and washed three times and then dried at 80 °C for 6 hours to get fine nanoparticles of AgInS_2_. Cu–AgInS_2_ and Mn–AgInS_2_ were prepared using a similar procedure by introducing 0.02 mmol of Cu and Mn while adjusting the Ag precursor to 0.18 mmol. For Cu and Mn co-doped AgInS_2_ nanoparticles, 0.02 mmol of Cu, 0.02 mmol of Mn, and 0.16 mmol of Ag were used, keeping indium and thiourea concentrations constant. Fig. 1 illustrates the synthesis procedure.

**Fig. 1:**
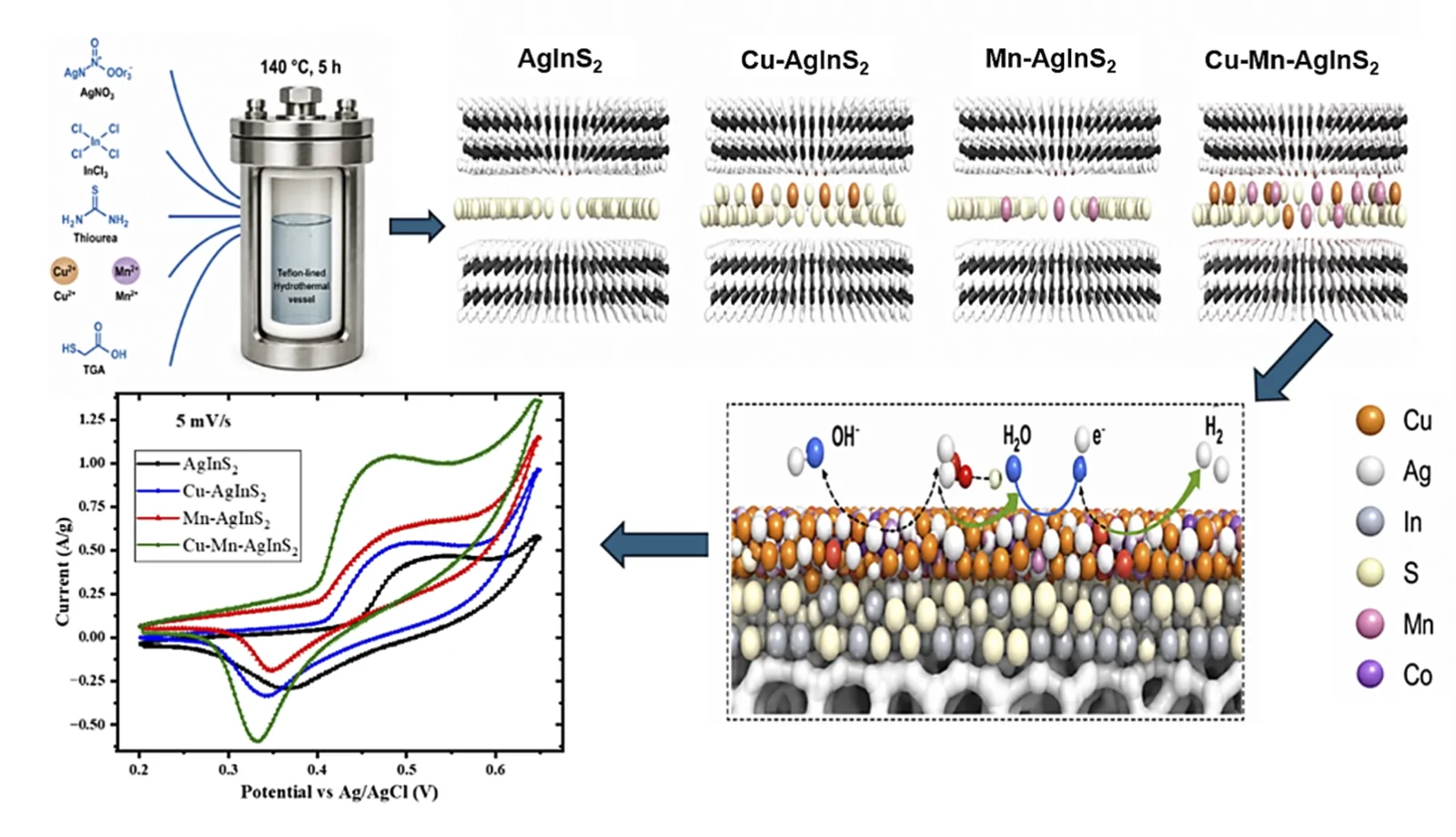
Schematic illustration of hydrothermal synthesis of AgInS_2_, Cu–AgInS_2_, Mn–AgInS_2,_ and Cu–Mn–AgInS_2_ nanoparticles.

### 2.3 Characterizations

Scanning electron microscope (SEM) was conducted using SU8600 from Japan HITACHI. In-Via Raman-PL setup (RENISHAW UK) using an Argon laser with an excitation wavelength of 457 nm. The absorption spectra of the NCs were obtained by employing a UV-VIS/NIR spectrometer LAMBDA750 (PerkinElmer). X-ray photoelectron spectroscopy (XPS) was conducted using a Thermo Scientific K-Alpha spectrometer under a vacuum better than 5 × 10^−7^ mbar, with a monochromatic Al Kα X-ray source operated at 12 kV and 6 mA. Electrochemical measurements, *i.e.*, cyclic voltammetry (CV), galvanostatic charge–discharge (GCD), and electrochemical impedance spectroscopy (EIS), were conducted on Origaflex 500. The synthesized material and activated carbon were dispersed in a solution of PVDF as the binder and DMF as solvent to make a slurry with a ratio of 70 : 20 : 10. Then, the slurry was deposited on nickel foam using the drop-casting method. CV was obtained at scan rates ranging from 5–100 mV s^−1^ within a potential window from 0.2–0.65 V, GCD was employed at various current densities of 0.22–0.67 A g^−1^, and EIS was used to analyze the electrode's impedance characteristics with an AC sine wave amplitude of 10 mV in a frequency range of 0.01 Hz to 100 kHz. All electrochemical measurements were performed in 2 M KOH by employing a three-electrode setup with Ag/AgCl as the reference, platinum wire as the counter, and the prepared material was deposited onto a current collector to fabricate the working electrode. The synthesized electrodes were employed as cathodic material for energy storage applications.

## 3. Results and discussions

### 3.1 Structural and morphological analysis

SEM and EDX analyses were done to observe the surface morphology of the prepared AgInS_2_ based chalcogenides before and after doping, and the results are displayed in Fig. 2. SEM micrographs of AgInS_2_, Cu-doped AgInS_2_, Mn-doped AgInS_2_, and co-doped Cu–Mn–AgInS_2_ chalcogenides are shown in Fig. 2(a–d). The compositional analysis confirms the presence of the expected constituent elements in the synthesized samples, without any additional elemental peaks, as shown in Fig. 2(e–h). The EDX spectra of AgInS_2_ confirm the presence of Ag, In, and S elements. Similarly, in the Cu-doped AgInS_2_ composite, additional Cu peaks were observed only to those that belong to the doped material, indicating successful incorporation of doped elements into the host material (*i.e.*, AgInS_2_). EDX spectrum of Mn-doped AgInS_2_ reveals the presence of Mn alongside Ag, In, and S, confirming successful integration of Mn. Similarly, in the case of the co-doped Cu–Mn–AgInS Chalcogenides, the spectra exhibit well-defined peaks of Cu and Mn in conjunction with the host material. Average particle size distribution histograms are displayed in Fig. 2(i–l).

**Fig. 2:**
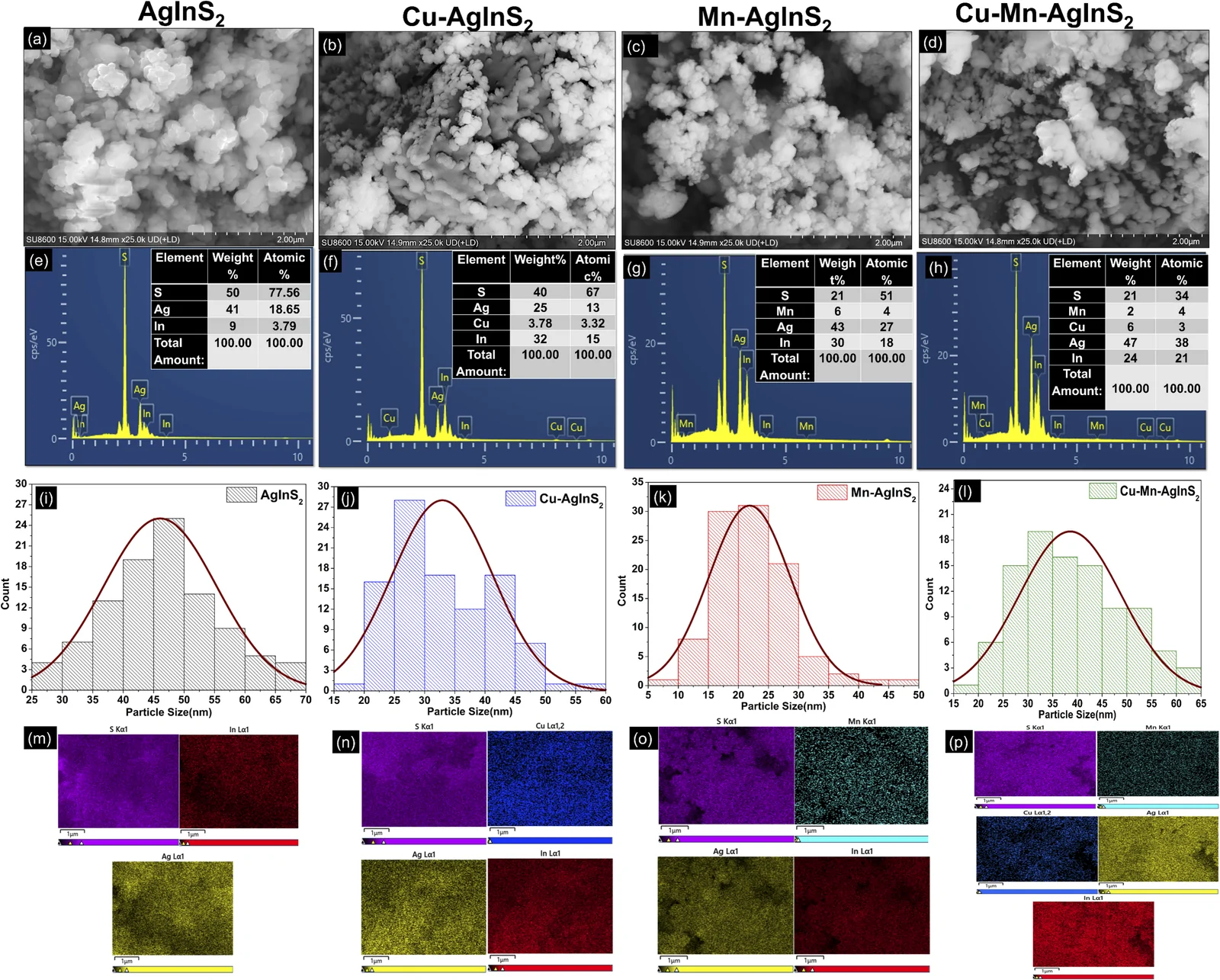
SEM micrographs (a–d), EDX spectra with elemental compositions (e–h), particle-size distributions with Gaussian fits (i–l), and elemental mapping images (m–p) of AgInS_2_, Cu–AgInS_2_, Mn–AgInS_2_, and Cu–Mn–AgInS_2_, respectively.

The pristine AgInS_2_ sample shows a rather broad distribution of particle sizes, with the sizes mostly in the range of about 40–55 nm. The doping of Cu and Mn leads to significant changes in the particle size and morphology. Cu-doped AgInS_2_ is shifted toward smaller particle sizes while Mn-doped AgInS_2_ exhibits the most significant reduction in size. The Cu–Mn co-doped sample demonstrates a broader particle size distribution than the individually doped samples and the distribution is shifted to smaller particle sizes as compared to pristine AgInS_2_. The observed particle size reduction with doping can be explained by the variation of AgInS_2_ nucleation and growth behavior. The addition of Cu and Mn can disturb the local lattice environment, altering the surface energy and growth kinetics, thus inhibiting particle growth and promoting the formation of smaller particles. The different particle-size distributions of the Cu-, Mn- and Cu–Mn-doped samples also suggest that the extent of this effect is dependent on the dopant composition.^23^ Moreover, the EDX elemental mapping shows a relatively uniform spatial distribution of the constituent elements including Cu and Mn in the analyzed regions of the corresponding doped samples without obvious elemental segregation, as shown in Fig. 2(m–p).

Raman spectra of AgInS_2_, Cu– AgInS_2_, Mn–AgInS_2_, and Cu–Mn–AgInS_2_ nanoparticles are displayed in Fig. 3. The first band indexed around 149–151 cm^−1^ is typically associated with *E*_g_(TO) vibrational mode of the Ag–S bond of AgInS_2_, and the second vibrational band, 215 cm^−1^ – 218 cm^−1^, is associated with the vibration of B_2_ transverse optical(TO) and E(TO) phonon mode^24^ respectively. The Raman band around 469–470 cm^−1^ in all samples is attributed to the second-order longitudinal optical (2LO) phonon vibrational mode corresponding to the S–S stretching mode that typically appears in metal chalcogenides.^25,26^

**Fig. 3:**
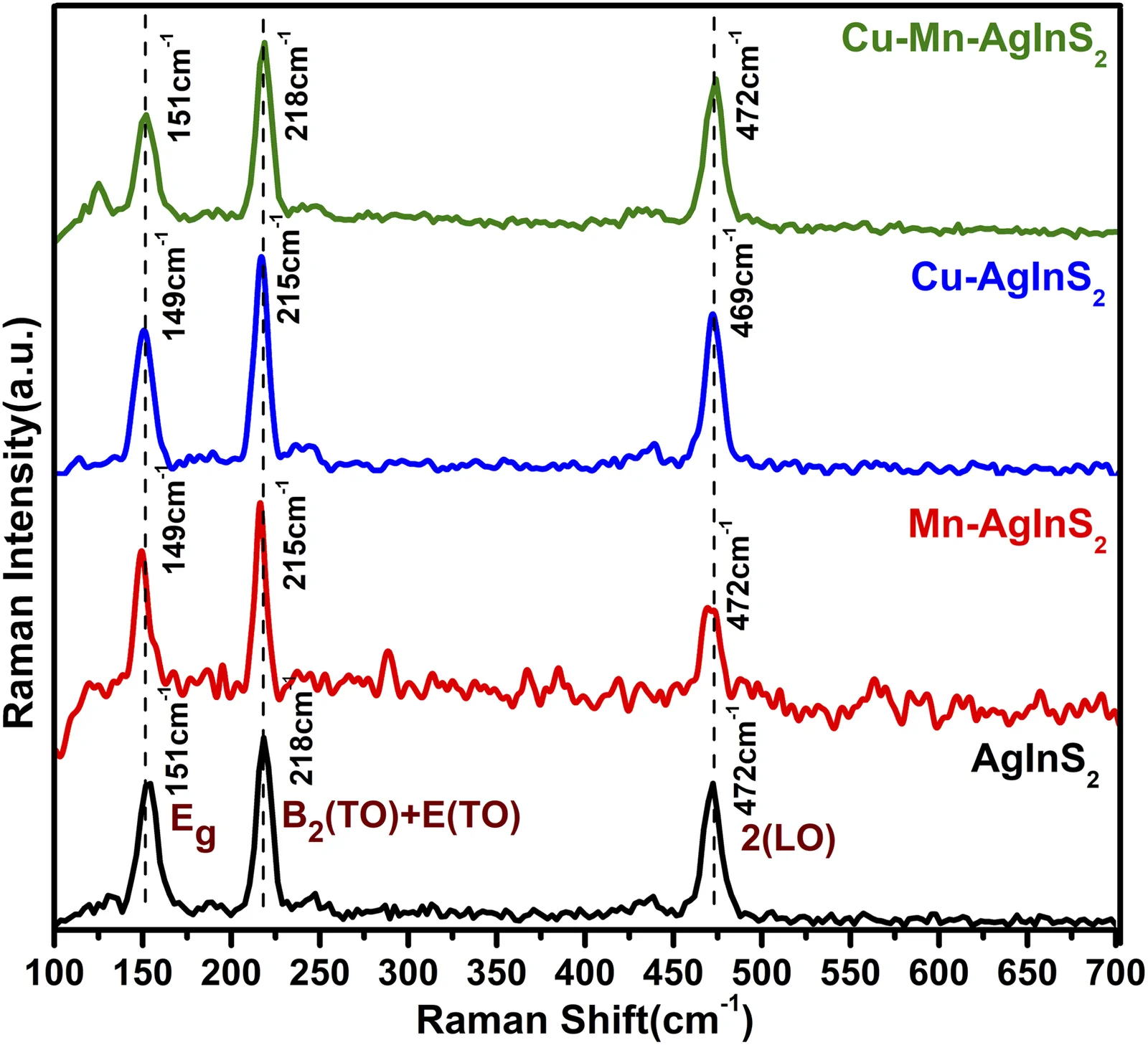
Raman spectra of the AgInS_2_, Cu–AgInS_2_, Mn–AgInS_2_ and Cu–Mn–AgInS_2_ nanoparticles.

The crystallographic structural analysis of the prepared samples was studied by utilizing X-ray diffraction (XRD) using X'Pert HighScore. The XRD patterns of all the prepared samples are depicted in Fig. 4.

**Fig. 4:**
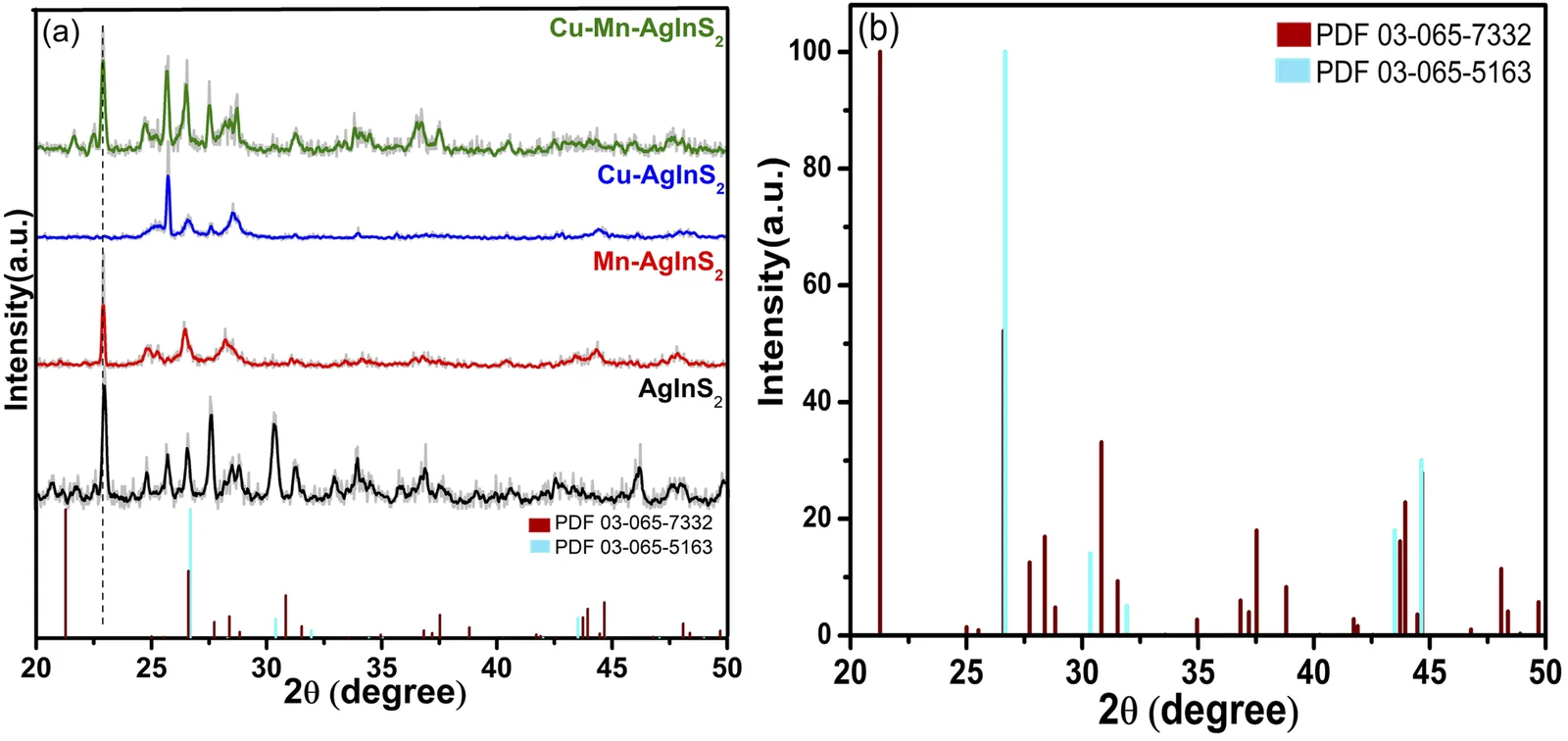
(a) XRD patterns of pristine AgInS_2_, Cu doped, Mn doped and Cu–Mn–AgInS_2_ and the standard reference reflections. (b) Standard reference patterns for orthorhombic AgInS_2_ (PDF No. 03-065-7332) and tetragonal AgInS_2_ (PDF No. 03-065-5163).

Fig. 4(a) shows the X-ray diffraction patterns of AgInS_2_, Cu–AgInS_2_, Mn–AgInS_2_ and Cu–Mn–AgInS_2_. For the pristine AgInS_2_ sample, the main reflections are located at around 2*θ* = 24.8°, 25.7°, 26.6°, 27.6°, 28.5°, 28.9°, 30.4°, 31.3°, 33.9°, 36.9° and 46.1°. The reflections are indexed as orthorhombic AgInS_2_ (PDF No. 03-065-7332) and tetragonal AgInS_2_ (PDF No 03-065-5163) Fig. 4(b), indicating a mixed-phase structure with orthorhombic phase as the dominant phase. The peak near 2*θ* = 23.0° may correspond to a minor secondary crystalline phase, in agreement with additional peaks reported for mixed-phase Ag–In–S materials.^27^ The preservation of orthorhombic and tetragonal AgInS_2_ in the doped samples indicates that incorporation of Cu and Mn preserves the overall host-phase assemblage but modifies its crystalline microstructure. The crystallite size of the synthesized nanocrystals was estimated using the Scherrer formula given in eqn (1).^28^1
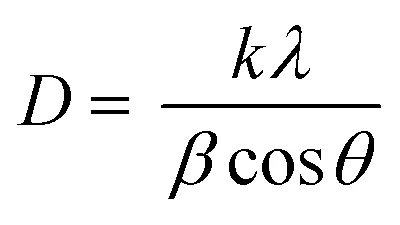
where *λ* ≈ 1.5406 Å is the wavelength of the Cu K alpha X-ray, while the Scherrer constant is denoted by *k* ≈ 0.9, and *β* represents the peak's full width half maximum. The estimated crystallite size is ≈ 25 nm, ≈14 nm, ≈9 nm and ≈18 nm for AgInS_2_, Cu–AgInS_2_, Mn–AgInS_2_, and Cu–Mn–AgInS_2_ respectively. Doping results in a decrease in crystallite size, suggesting changes in nucleation and crystal growth during hydrothermal synthesis. The smallest crystallite size is observed for Mn–AgInS_2_. The co-doped sample has larger crystallite size than each of the singly doped samples, but smaller crystallite than the undoped AgInS_2_, implying that the incorporation of both Cu and Mn influences crystal growth differently from individual doping.

The XRD results of crystallite sizes are smaller than the SEM results of particle sizes. This is because XRD measures the size of coherently diffracting crystalline domains while SEM measures the physical dimensions of the particles. Thus, a particle observed in SEM may be composed of one or more crystallites.^29^ Despite this, both techniques reveal a consistent size change upon doping, influence the role of Cu and Mn on the growth of AgInS_2_.

The presence of all expected elements: Ag, In, S, Cu, and Mn along with adventitious C and O. The quantitative analysis from the survey spectra is summarized in Table (inset in survey graph). The elemental composition and chemical states of the synthesized Cu–Mn–AgInS_2_ material were investigated using X-ray photoelectron spectroscopy (XPS). The survey spectrum confirmed the high-resolution C 1s spectrum (Fig. 5(a)) was used for charge correction, with the adventitious carbon peak calibrated to 284.8 eV. The spectrum was fitted using a smart background subtraction and Gaussian–Lorentzian peak shapes. The deconvoluted C 1s spectrum reveals four distinct components corresponding to different carbonaceous species. The dominant peak at 284.8 eV (FWHM = 1.25 eV, 77.77 at%) is attributed to the C–C/C–H bonds from adventitious surface carbon. The peak at 285.6 eV (13.37 at%) is assigned to C–N/C–OH species, while the component at 286.4 eV (2.47 at%) corresponds to C–O–C (ether/alcohol) linkages. A minor peak observed at 289.3 eV (6.38 at%) is characteristic of O–C=O (carboxylate or carbonate) groups. The ratio of non-oxidized (C–C/C–H) to oxidized carbon species was determined to be approximately 3.5 : 1 (77.77% *vs.* 22.23%), indicating a clean surface with only minor adventitious oxidation. The presence of these oxygenated functionalities is typical for nanomaterials exposed to ambient conditions, indicating a mild degree of surface oxidation or residual organic species from the synthesis, while the strong C–C peak confirms the calibration reliability and overall sample stability.

**Fig. 5:**
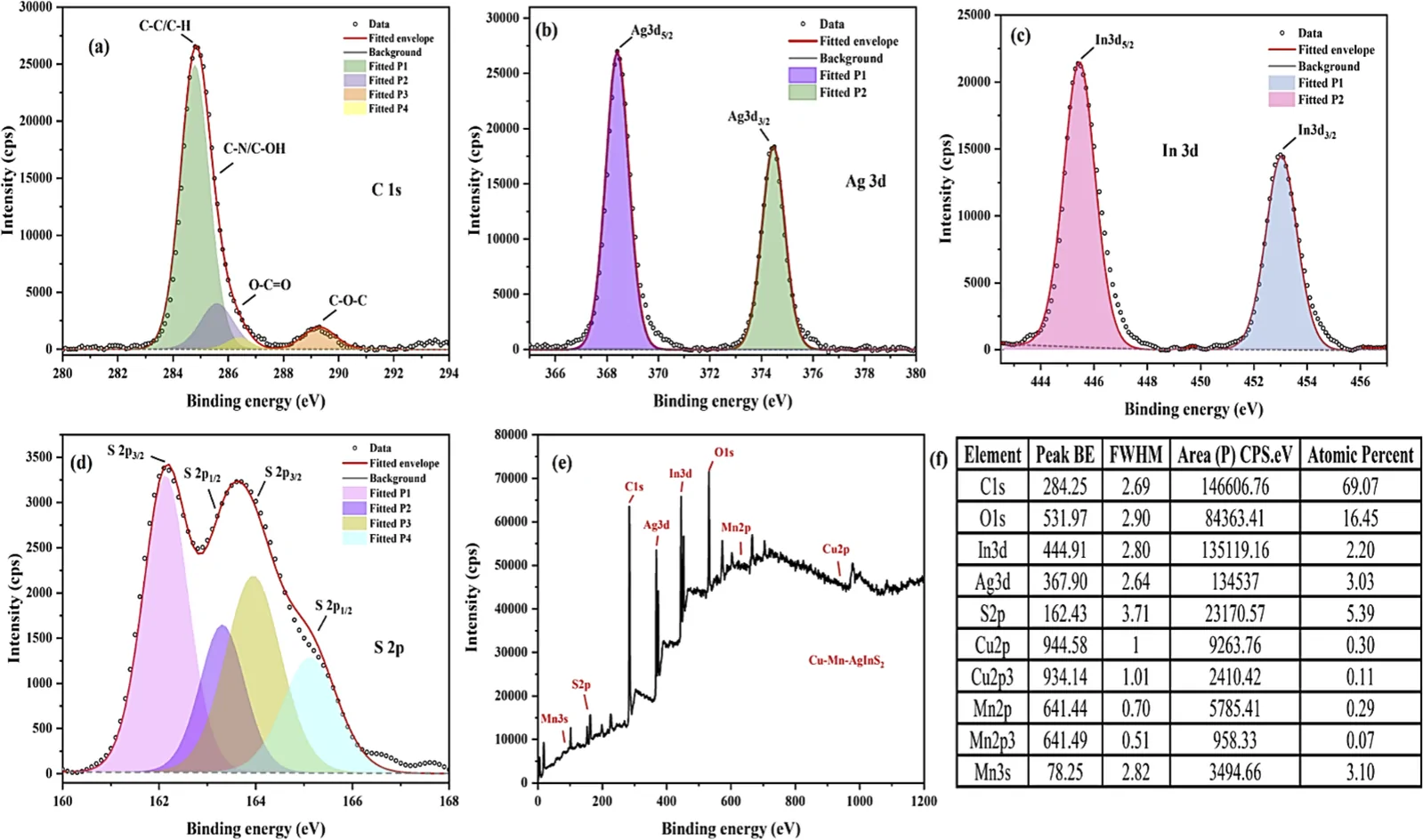
XPS spectra of Cu–Mn–AgInS_2_: (a) C 1s spectrum. (b) Ag 3d spectrum showing Ag^+^ (Ag 3d_5/2_ at 368.41 eV). (c) In 3d spectrum showing In^3+^ (In 3d_5/2_ at 445.45 eV). (d) S 2p spectrum fitted with two doublets (area ratio 2 : 1, splitting 1.18 eV): dominant S^2−^ at 162.13 eV and minor elemental sulfur/C–S at 163.95 eV. (e) Wide range survey spectrum confirming the presence of Ag, In, S, Cu, and Mn. All spectra were charge-corrected to the adventitious C 1s peak at 284.8 eV. (f) Binding energies, full widths at half maximum (FWHM), peak areas and atomic percentages from XPS analysis.

The high-resolution Ag 3d spectrum (Fig. 5(b)) exhibits a single spin–orbit doublet with Ag 3d_5/2_ and Ag 3d_3/2_ peaks at binding energies of 368.41 eV and 374.46 eV, respectively. The spin–orbit splitting of 6.05 eV closely matches the theoretical value of 6.0 eV, confirming that silver is exclusively present in the +1-oxidation state (Ag^+^). The integrated area ratio of 1.472 : 1 is in excellent agreement with the theoretical 3 : 2 ratio, confirming a single chemical environment without metallic Ag^0^ or other silver-containing impurities. The FWHM values of the Ag 3d_5/2_ and Ag 3d_3/2_ peaks are 1.06 eV and 1.05 eV, respectively, demonstrating excellent crystallinity and a well-fitted spectrum. These results confirm the successful incorporation of silver into the AgInS_2_ host lattice

The high-resolution In 3d spectrum (Fig. 5(c)) exhibits a single spin–orbit doublet with In 3d_5/2_ and In 3d_3/2_ peaks at binding energies of 445.45 eV and 453.03 eV, respectively. The spin–orbit splitting of 7.58 eV closely matches the theoretical value of 7.56 eV, confirming that indium is exclusively in the +3-oxidation state (In^3+^). The integrated area ratio of 1.485 : 1 is in excellent agreement with the theoretical 3 : 2 ratio, confirming a single chemical environment without metallic In^0^. The FWHM values of the In 3d_5/2_ and In 3d_3/2_ peaks are 1.40 eV and 1.39 eV, respectively, demonstrating excellent crystallinity and a clean, well-fitted spectrum. These results confirm the successful incorporation of indium into the AgInS_2_ host lattice. The high-resolution S 2p spectrum (Fig. 7(d)) was fitted with two spin–orbit doublets, constraining the S2p_3/2_ : S2p_1/2_ area ratio to 2 : 1 and the spin–orbit splitting to 1.18 eV. The dominant doublet, with S 2p_3/2_ at 162.13 eV and S 2p_1/2_ at 163.31 eV, is attributed to the sulfide (S^2−^) species of the AgInS_2_ host lattice, accounting for approximately 53.8% of the total sulfur signal. A second doublet, with S 2p_3/2_ at 163.95 eV and S 2p_1/2_ at 165.13 eV, is assigned to elemental sulfur or C–S bonded species, accounting for approximately 46.2% of the total sulfur signal. The integrated area ratios (2.021 : 1 and 1.709 : 1) are in good agreement with the theoretical 2 : 1 ratio, confirming the reliability of the fit. The FWHM values of both doublets were constrained to be equal (1.10 eV for sulfide and 1.35 eV for elemental sulfur), ensuring physical consistency. No sulfate species (SO_4_^2−^) were detected at ∼168.5 eV, indicating good surface stability against oxidation. These results confirm the successful formation of the desired sulfide structure, with only minor residual organic sulfur species. The XPS survey spectrum (Fig. 5(e)), charge-corrected using the adventitious C 1s peak at 284.8 eV, confirmed the presence of Ag, In, S, Cu, and Mn, along with adventitious C and O. The Ag 3d_5_/_2_ and In 3d_5/2_ peaks at 367.90 eV and 444.91 eV correspond to Ag^+^ and In^3+^, respectively, while the S 2p peak at 162.43 eV confirms the sulfide (S^2−^) species. The Cu 2p_3/2_ peak at ∼934.14 eV and the Mn 2p_3_/_2_ peak at 641.49 eV confirm the presence of Cu and Mn dopants, with total atomic percentages of 0.41 at% and 0.36 at%, respectively. The Mn 2p_3_/_2_ binding energy (641.49 eV) and the Mn 3s multiples splitting of ∼5.6 eV is characteristic of the Mn^2+^ oxidation state. The Cu 2p_3/2_ peak at ∼934.14 eV is consistent with Cu^+^, though the weak signal (total 0.41 at%) precludes detailed peak fitting. The low Cu and Mn signals are attributed to their intended low dopant concentrations. These results confirm the successful incorporation of Cu and Mn dopants into the AgInS_2_ host lattice without altering the primary oxidation states of Ag^+^, In^3+^, and S^2−^. These XPS results confirm the successful synthesis of Cu–Mn–AgInS_2_ with the desired elemental composition and oxidation states, validating the material's potential for further investigation in energy storage applications.

### 3.2 Absorption and photoluminescence (PL) spectroscopy analysis

The UV-vis spectra as shown in Fig. 6 have been obtained in the 250–700 nm region. The linear part of (*αhv*)^2^*vs. hν* is extrapolated to zero to determine the optical band gap for the absorption peak.

**Fig. 6:**
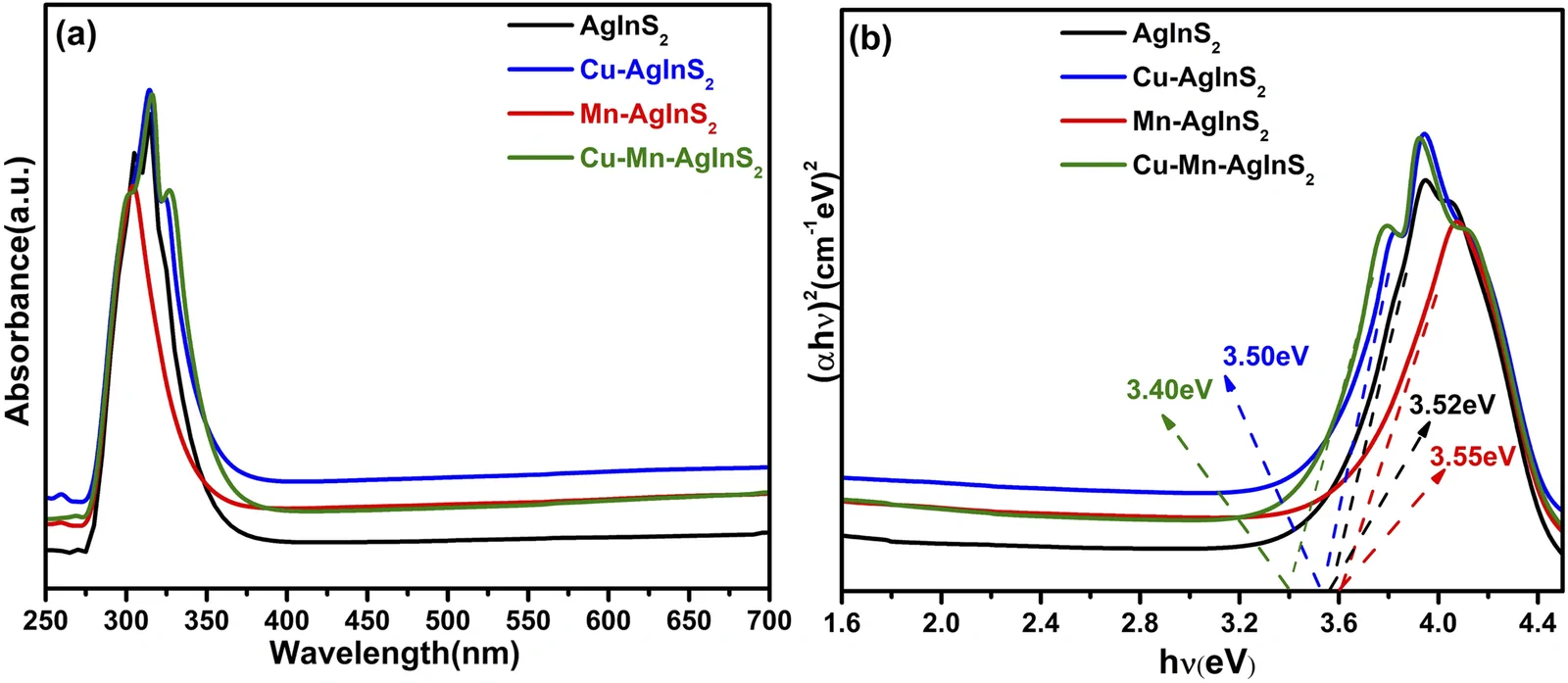
(a) UV-visible absorption spectra. (b) Tauc Plots of AgInS_2_, Cu–AgInS_2_, Mn–AgInS_2_, and Cu–Mn–AgInS_2._

The band gap of all the samples is estimated using the Tauc equation, where “*α*” is the absorption coefficient, *h* is Planck's constant, *ν* is the frequency, and *E*_g_ is the optical band gap of the material.^30^

2 *ahv* = *A*(*hv* − *E*_g_)^*n*^

The estimated band gaps for the AgInS_2_, Cu–AgInS_2_, Mn–AgInS_2_, and Cu–Mn–AgInS_2_ are ∼3.52 eV, 3.50 eV, 3.55 eV, and 3.40 eV, respectively. The small band gap in the case of Cu–Mn co-doped AgInS_2_ might be due to strain effects, dopant-induced localized energy levels, and stronger band tailing that can shift the absorption edge towards lower energies. While Mn doping slightly increases the band gap of the AgInS_2_ NCs might be due to the quantum confinement effect. Overall, the Cu and Mn co-doping in AgInS_2_ strongly modify the electronic state and show the red shift in the absorption band edge and have the comparatively smallest optical band gap among all four samples.

PL spectroscopy was employed to study the defect states. The PL spectra of all as-synthesized nanoparticles are depicted in Fig. 7. PL spectra of AgInS_2_, Cu–AgInS_2_, Mn–AgInS_2_, and Cu–Mn–AgInS_2_ exhibit the broad emission hump spanning 500–900 nm, indicating the presence of the intrinsic point defects (vacancies and interstitials) and dopant-induced defect states within the band structure.

**Fig. 7:**
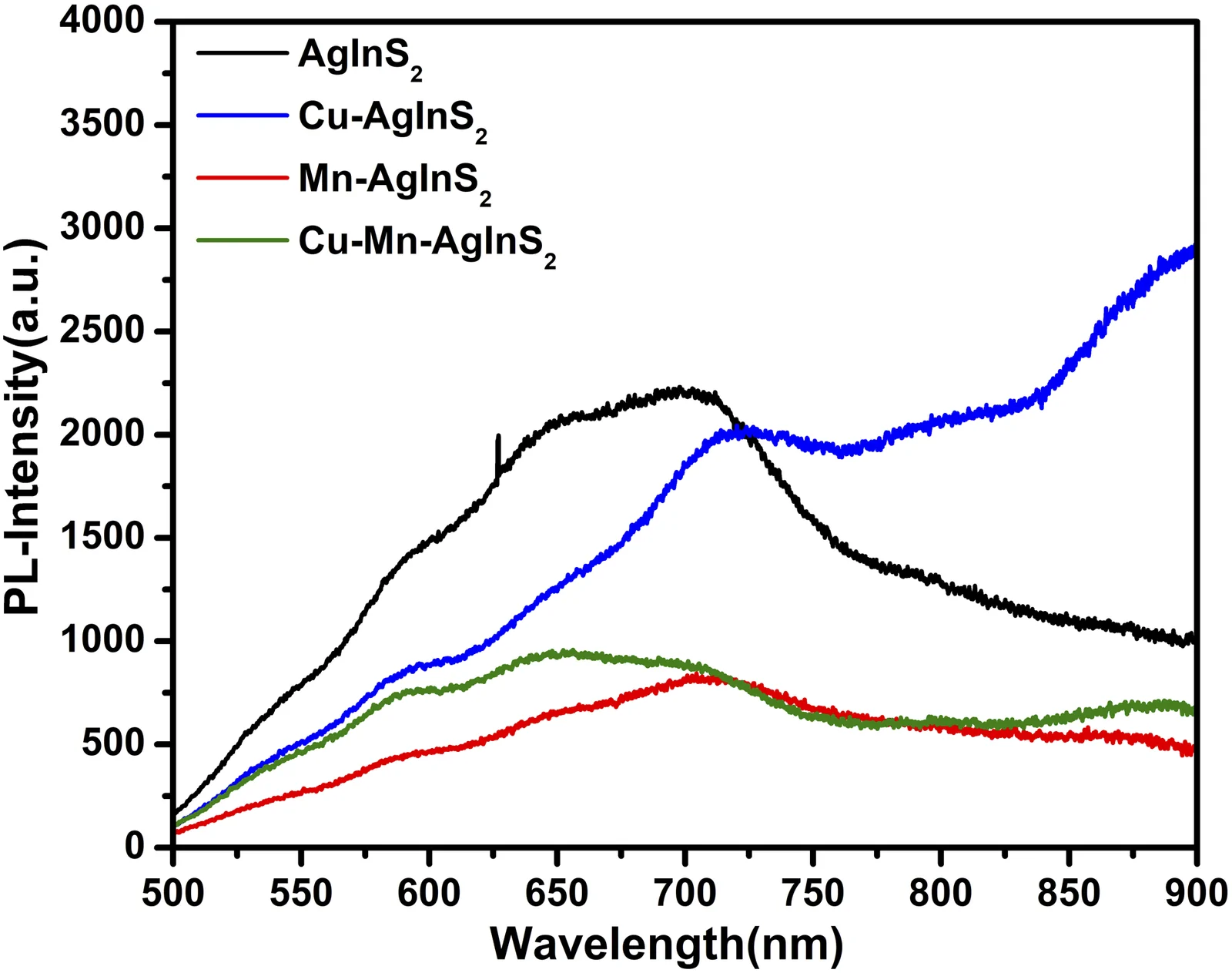
PL spectra of AgInS_2_, Cu–AgInS_2_, Mn–AgInS_2_ and Cu–Mn–AgInS_2._

From the perspective of supercapacitor applications, the defect states play a vital role in regulating charge transfer processes and charge storage. The pristine AgInS_2_ exhibited a broad emission spectrum centered around 700 nm, attributed to the donor–acceptor pair (DAP) recombination and intrinsic defects related to sulfur (S) and silver (Ag)/indium (In)-related defect vacancies/interstitials. These defect states can serve as electrochemical active sites. Cu doping in AgInS_2_ introduces deep-level defect (DLD) states that improve carrier density and electron hopping and increase the electrical conductivity, thereby enhancing the pseudocapacitive charge storage. In contrast, the Mn doping reduces the carrier PL intensity with emission bands around 596, 701, and 809 nm, indicating reduced charge recombination and efficient charge separation, which is suitable for rapid electron transport during electrochemical cycling. The co-doped Cu–Mn–AgInS_2_ sample shows overall lower emission compared to Cu–AgInS_2_ and Mn–AgInS_2_, suggesting a synergistic interaction of Cu and Mn-induced defect states. The defect engineering creates multiple charge transfer pathways to increase the electrochemical active sites and reduce the recombination losses that result in improved electrical conductivity, enhanced charge transfer kinetics, greater ion accessibility, and superior pseudocapacitive behavior.

### 3.3 Electrochemical performance

Fig. 8a depicts the cyclic voltametric results of the prepared electrodes at 5 mV s^−1^ over a potential window of 0.2–0.65 V. The prepared electrodes exhibit prominent redox peaks indicating the pseudocapacitive behavior of the electrodes. The observed difference between the oxidation and reduction peaks is attributed to fast ion transport. When comparing AgInS_2_, Cu–AgInS_2_, Mn–AgInS_2_, and Cu–Mn–AgInS_2_, a significant increase in current and area under the curve is observed for the samples with doping. The prepared electrode's electrochemical activity enhances considering the trend AgInS_2_ < Cu–AgInS_2_ < Mn–AgInS_2_ < Cu–Mn–AgInS_2_. Because of its lower conductivity and fewer accessible active sites, pristine AgInS_2_ exhibits the lowest current response. This implies that the efficiency of charge storage and redox kinetics can be improved by doping with copper and manganese species. Fig. 8(b) represents the specific capacitance obtained at different scan rate obtained using cyclic voltammetry. The maximum specific capacitance obtained at 5 mV s^−1^ by comparing AgInS_2_, Cu–AgInS_2_, Mn–AgInS_2_, and Cu–Mn–AgInS_2_ is 72.3, 96.7, 129.8, 189.3 F g^−1^, respectively. The maximum specific capacitance is exhibited by dual-doped Cu–Mn–AgInS_2_ sample, *i.e.*, 189.3 F g^−1^, indicating a substantial boost in electrochemical activity when compared with pristine AgInS_2_. The specific capacitance of the prepared electrode is represented in tabular form (Table 1).

**Fig. 8:**
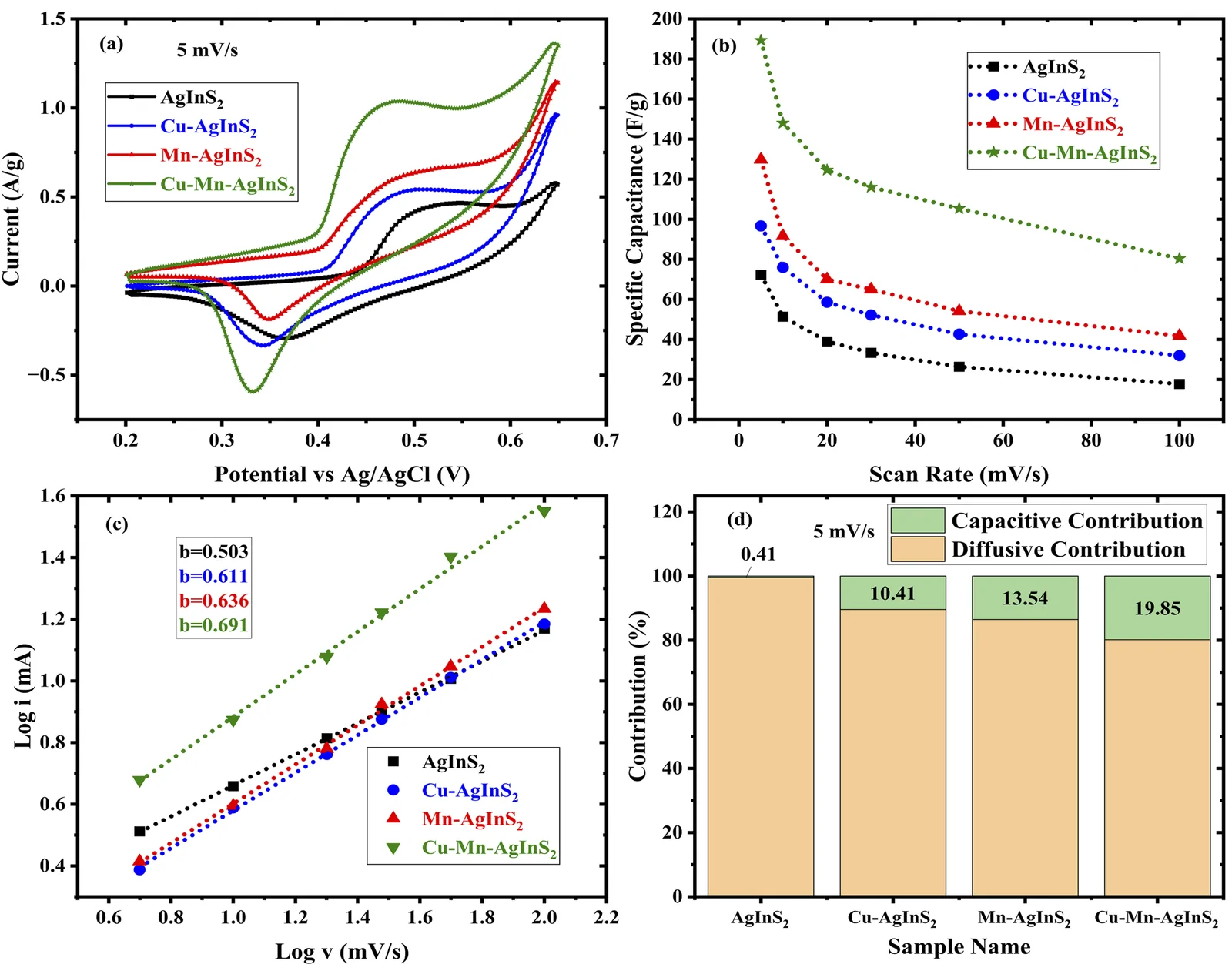
(a) CV curve at 5 mV s^−1^, (b) specific capacitance at various scan rates, (c) linear fitting of log *i vs.* log *v*, (d) contribution analysis at 5 mV s^−1^ of AgInS_2_, Cu–AgInS_2_, Mn–AgInS_2_, and Cu–Mn–AgInS_2._

**Table 1:** Specific capacitance values of the prepared electrode at various scan rates

Scan rate (mV s^−1^)	Specific capacitance (F g^−1^)			
	AgInS_2_	Cu–AgInS_2_	Mn–AgInS_2_	Cu–Mn–AgInS_2_
5	72.31	96.68	129.80	189.27
10	51.36	75.97	91.60	148.00
20	39.01	58.60	70.05	124.53
30	33.33	52.19	65.00	116.01
50	26.39	42.68	54.15	105.40
100	17.77	31.96	41.79	80.33

To further understand the electrochemical properties of pristine AgInS_2_ and doped-AgInS_2_ electrodes, the contributions were calculated, as shown in Fig. 8(d). The relation between peak current (*i*) and scan rate (*v*) is determined using the power-law equations.^31^

3 *i* = *av*^*b*^

In the equation, *a* and *b* are constants, *i* is the current at the peak (mA), and *v* is the scan rate (mV s^−1^). The slope of the plot of log(*i*) *vs.* log(*v*) gives the value of *b*, which is a significant indicator of the charge storage mechanism.

4 *i* = log(*a*) + *b* log(*v*)

If *b* = 0.5, it denotes a diffusion-controlled faradaic intercalation mechanism, while a *b*-value of 1.0 implies a surface-controlled capacitive process. The computed *b*-values are depicted in Fig. 8(c), range from 0.5 to 0.69, displaying a hybrid charge storage behavior that combines diffusion- and capacitive-controlled mechanisms. Additionally, the capacitive contribution may be calculated by employing eqn (5).^32^

5 *i*(*v*) = *k*_1_*v* + *k*_2_*v*^1/2^

where *k*_1_*v* and *k*_2_*v*^1/2^ denote the contributions of capacitive and diffusion-controlled mechanisms, respectively. Fig. 8(d) demonstrates contribution behaviors at 5 mV s^−1^. The capacitive contribution ratio for pristine AgInS_2_ grows from 0.41 to 1.82% as the scan rate increases. The introduction of the dopant also increases the capacitive contribution from 0.41% (pristine AgInS_2_) to 10.41% (Cu–AgInS_2_), 13.54% (Mn–AgInS_2_), and 19.85% (Cu–Mn–AgInS_2_) for 5 mV s^−1^.

Fig. 9(a) shows the galvanostatic discharge curves of the pristine AgInS_2_ and doped AgInS_2_. All the electrodes exhibit non-linear and plateau-like shapes, which indicates that the charge storage process occurs mainly through faradaic pseudocapacitive redox processes and not just through electrostatic double-layer capacitance. The pristine AgInS_2_ exhibits the shortest discharge time of about 66.6 s. The discharge times of the Cu-doped and the Mn-doped samples are significantly extended to about 96.4 s for Cu–AgInS_2_ and 139.46 s for Mn–AgInS_2_. However, the greatest discharge time of about 228.82 s exhibit by the co-doped electrode *i.e.*, Cu–Mn AgInS_2_. This superior performance arises from the strong synergistic effects of Cu and Mn ions, which enable optimized electron transport kinetics, enhance the electroactive surface area, and enable multi-electron redox reactions. The specific capacitance of the prepared electrodes is obtained at various current densities (0.22 A g^−1^ to 0.67 A g^−1^). The pristine AgInS_2_ showed the maximum specific capacitance of 33.49 F g^−1^, which is improved by doping with Cu (48.94 F g^−1^) and Mn (69.96 F g^−1^), and with dual doping taking advantage of both Cu and Mn, Cu–Mn– AgInS_2_ exhibited 115.58 F g^−1^. The prepared electrodes exhibit the maximum specific capacitance at 0.22 A g^−1^. A decline in specific capacitance was seen as current density increased, which can be ascribed to the limited availability of the active material for redox reactions at high current density. Fig. 9(c) depicts the Ragone plot of the prepared electrode, and the Cu–Mn–AgInS_2_ depicts the energy density of 3.11 Wh kg^−1^ at 48.88 W kg^−1^ power density, which is approximately 3.4 times greater than the pristine AgInS_2_ (Table 2).

**Fig. 9:**
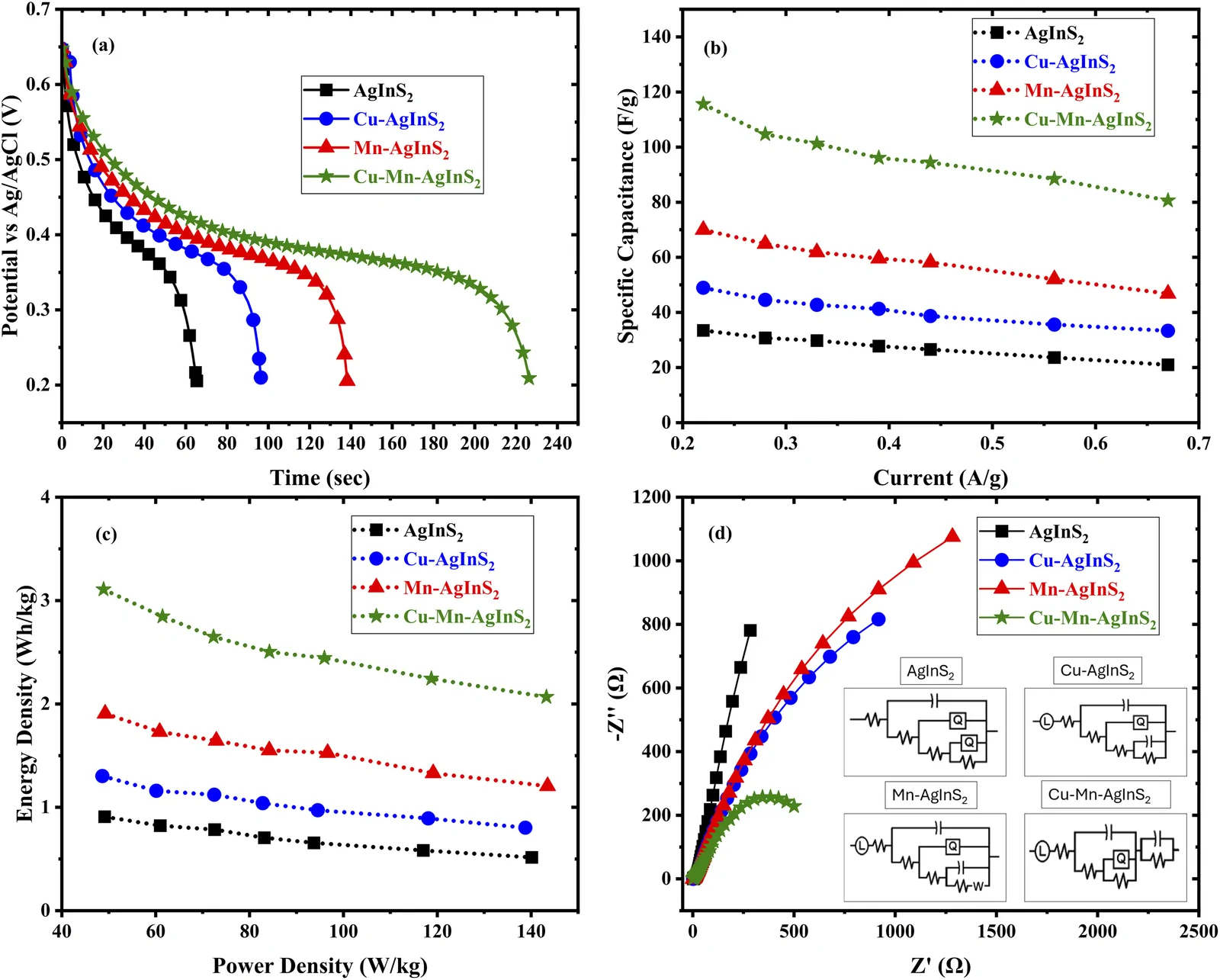
(a) GCD at 0.22 A g^−1^, (b) specific capacitance at different current densities, (c) Ragone plot (d) Nyq *Z* plot of AgInS_2_, Cu–AgInS_2_, Mn–AgInS_2_, and Cu–Mn–AgInS_2_.

**Table 2:** Specific capacitance, energy density, and power density of the prepared electrode obtained at various current densities

Electrodes	Current density (A g^−1^)	Specific capacitance (F g^−1^)	Energy density (Wh kg^−1^)	Power density (W kg^−1^)
AgInS_2_	0.22	33.49	0.91	49.11
	0.28	30.71	0.82	60.96
	0.33	29.77	0.78	72.55
	0.39	27.76	0.71	83.14
	0.44	26.54	0.66	93.71
	0.56	23.65	0.58	117.02
	0.67	20.99	0.52	140.12
Cu–AgInS_2_	0.22	48.94	1.30	48.63
	0.28	44.55	1.16	60.13
	0.33	42.72	1.12	72.46
	0.39	41.28	1.04	82.81
	0.44	38.66	0.97	94.53
	0.56	35.56	0.89	118.08
	0.67	33.33	0.80	138.77
Mn–AgInS_2_	0.22	69.96	1.91	49.22
	0.28	64.93	1.73	60.83
	0.33	61.84	1.64	72.91
	0.39	59.57	1.55	84.19
	0.44	58.15	1.53	96.61
	0.56	52.05	1.33	119.11
	0.67	46.82	1.21	143.50
Cu–Mn–AgInS_2_	0.22	115.58	3.11	48.88
	0.28	104.66	2.85	61.45
	0.33	101.25	2.65	72.33
	0.39	96.07	2.50	84.22
	0.44	94.35	2.44	95.95
	0.56	88.45	2.24	118.71
	0.67	80.60	2.07	143.25

Fig. 9(d) depicts the Nyquist *z* plot, which was employed for investigating charge transfer kinetics of prepared electrodes. A semicircle in the high-frequency region shows charge-transfer resistance (*R*_ct_) at the electrode–electrolyte interface. An intercept on the real axis in the high-frequency region represents solution resistance; a small *R*_s_ indicates the electrolyte's excellent conductance of ions. AgInS_2_ largest semicircle indicates relatively low electrical conductivity and the highest charge-transfer resistance (*R*_ct_). The pristine AgInS_2_ exhibits solution resistance *R*_s_ of 2.45 Ω and charge-transfer resistance *R*_ct_ of 1.652 Ω. Upon the integration of Cu, Cu–AgInS_2_ has a much smaller semicircle diameter than AgInS_2_, indicating a significantly lower *R*_ct_ of 1.3 Ω and better interfacial charge-transfer kinetics. The significantly smaller semicircle of Cu–AgInS_2_ indicates that Cu injection improves electron mobility and interfacial charge transfer. The solution resistance of Cu–AgInS_2_ is 2.065 Ω. The lower impedance shows that Cu doping increases electrical conductivity and encourages redox reactions. This demonstrates how Cu addition significantly enhances the ionic and electrical transport properties of AgInS_2_. However, when doping of manganese was done with AgInS_2_ the sample exhibits solution resistance of *R*_s_ is 2.172 Ω and the charge-transfer resistance *R*_ct_ of 1.22 Ω, which is due to faster ion diffusion pathways and increased redox activity caused by Mn doping. The co-doping of Cu and Mn with AgInS_2_ exhibits the smallest semicircle of all the samples, indicating the lowest *R*_ct,_*i.e.*, 0.693 Ω, and exceptionally fast charge-transfer processes. In addition, the minimum *R*_s_ of 1.776 Ω also proves the strong electrolyte compatibility. The co-doping of Cu and Mn gives a synergistic effect, increasing the active site density and conductivity at the interface, which in turn increases the interfacial kinetics. The virtually vertical tail of the low-frequency region indicates highly efficient ion diffusion, which characterizes a perfect capacitive response. Cu–Mn–AgInS_2_ shows the best overall electrochemical impedance performance, highlighting the effectiveness of dual-doping in optimizing AgInS_2_-based electrode materials.

The lower charge-transfer resistance *R*_ct_ of the Cu or Mn-doped AgInS_2_ electrode suggests that electron transfer at the electrode/electrolyte interface is more facile. This improvement can be owed to the modification of the electronic structure caused by the incorporation of Cu and Mn in the AgInS_2_ lattice. The incorporation of Cu has been reported to induce Cu 3d localized electronic states in AgInS_2_, which alters the electronic structure of the material and the localization of charge carriers.^33^ Likewise, Mn incorporation can affect the local electronic environment of the AgInS_2_ and impact on its charge-carrier dynamics.^34^ Therefore, simultaneous doping with both Mn and Cu has been reported to result in a synergistic electronic effect in AgInS_2_-based materials that facilitates charge transfer.^35^

The electronic modification induced by Cu or Mn in the AgInS_2_ could ease the charge redistribution and result in better charge transport paths in the AgInS_2_ system and at the electrode/electrolyte interface. Thus, the lower *R*_ct_ observed and the smaller Nyquist semicircle for Cu or Mn-doped AgInS_2_ electrode can be correlated with lower interfacial resistance and better charge transfer kinetics (Table 3).

**Table 3:** Solution and charge transfer resistance of prepared electrodes

Electrodes	Solution resistance, *R*_s_ (Ω)	Charge transfer resistance, *R*_ct_ (Ω)
AgInS_2_	2.45	1.652
Cu–AgInS_2_	2.065	1.3
Mn–AgInS_2_	2.172	1.22
Cu–Mn–AgInS_2_	1.776	0.693

## 4. Conclusion

In the present study, a facile hydrothermal method was employed to fabricate AgInS_2_ and Cu-, Mn-, and Cu–Mn-doped AgInS_2_ nanoparticles. Structural and spectroscopic analysis confirmed that the incorporation of Cu and Mn altered the crystal structure, morphology, and electronic properties of AgInS_2_. Co-doping of Cu–Mn is an efficient route to engineer the structural, electronic, and electrochemical properties of AgInS_2_ based chalcogenides. The doping of Cu and Mn changes the crystal structure and defect landscape of AgInS_2_ and provides favorable pathways for electrochemical charge storage. More importantly, the co-doping of Cu and Mn brings about a synergistic effect to improve the redox activity, interfacial charge transfer, ion transport, and the accessibility of electroactive sites. Therefore, the pseudocapacitive performance of the co-doped material is greatly improved compared to AgInS_2_ and the single-doped ones. The results demonstrate a direct correlation between the dopant-induced defect engineering and enhanced electrochemical kinetics, thus emphasizing the significance of rational multication engineering in optimizing AgInS_2_-based electrodes. The best results are demonstrated by the dual-doped Cu–Mn–AgInS_2_, depicting the highest electrochemical performance, with a specific capacitance of 189.27 F g^−1^ at 5 mV s^−1^ and 115.58 F g^−1^ at 0.22 A g^−1^, an energy density of 3.11 Wh kg^−1^ at a power density of 48.88 W kg^−1^ and 2.07 Wh kg^−1^ at a power density of 143.25 W kg^−1^. The sample also exhibits minimum solution resistance of 1.776 Ω and a charge transfer resistance of 0.693 Ω. This work offers a promising materials-design approach to enhance the charge-storage characteristics of chalcogenide-based electrodes for next-generation supercapacitor applications.

## Conflicts of interest

The authors declare no conflicts of interest.

## Data Availability

The experimental data supporting the conclusions of this study are presented within the manuscript. No additional data are available.
